# Integrated Full-Length Transcriptome and Metabolome Profiling Reveals Flavonoid Regulation in Response to Freezing Stress in Potato

**DOI:** 10.3390/plants12102054

**Published:** 2023-05-22

**Authors:** Zhiguo Zhu, Lingling Wei, Lei Guo, Huihui Bao, Xuemei Wang, Philip Kear, Zhen Wang, Guangtao Zhu

**Affiliations:** 1 Yunnan Key Laboratory of Potato Biology, Engineering Research Center of Sustainable Development and Utilization of Biomass Energy, Ministry of Education, School of Life Sciences, Yunnan Normal University, Kunming 650500, China; 2 School of Life Sciences, Anhui Agricultural University, Hefei 230036, China; 3 International Potato Center (CIP), CIP China Center for Asia Pacific, Beijing 100081, China

**Keywords:** *S. commersonii*, freezing stress, full-length transcriptome, metabolome, flavonoid metabolic pathway, transcription factor

## Abstract

Cold stress impairs plant growth and development, resulting in crop failure. Cultivated potato (*Solanum tuberosum* L.) is sensitive to freezing, while its wild relative, *S. commersonii*, has a strong freezing tolerance. To decipher the anti-freezing mechanism of CM, we carried out a transcriptomic and metabolomic analysis of an anti-freezing variety of CM (a type of *S. commersonii*) and a freeze-sensitive variety of DM (a type of *Solanum tuberosum* L.). A total of 49,232 high-quality transcripts from 12,811 gene loci, including 46,772 coding sequences and 2018 non-coding RNAs, were identified. KEEG enrichment analysis of differentially expressed genes (DEGs) between the two varieties showed that the flavonoid biosynthesis pathway was strongly induced by freezing stress, which was proven by flavonoid metabolome analysis. Consistent with the accumulation of more flavonoids, nearly all the pathway genes were significantly upregulated in CM than those in DM. The transcript levels of two *chalcone synthase* (*CHS*-*1*) isoforms and four isoforms of *flavonoid 3′-hydroxylase* (*F3′H*-*1*) were confirmed by qRT-PCR. Co-expression analysis identified one *Myb-related* and three *UGT*s (*UDP*-*glycosyltransferase*) that were significantly upregulated in CM during freezing stress. Our findings support that the flavonoid pathway was significantly enhanced by freezing stress and the greater accumulation ofglycosylatedflavonoids in resistant types than that of sensitive types, maybe accounting for the increased freezing tolerance of freeze-resistant potato varieties.

## 1. Introduction

The potato (*Solanum tuberosum* L.) is one of the most important tuber crops in the world, playing an important role in ensuring food security. The cultivated potato variety is sensitive to frost, exhibiting chilling damage at −0.8 °C and frost damage at −2 °C [1,2]. Even a brief exposure to cold stress can substantially reduce potato production [3]. The genetic improvement of the plant to improve freezing tolerance is the most effective way to address this problem.

Plants respond to environmental stimuli by regulating gene expression, and transcriptome analysis is an important method to investigate this process. Therefore, the acquisition of intact transcripts is a prerequisite for functional genomic [4]. Single-molecule real-time (SMRT) sequencing, a third-generation sequencing technique, can read >10 kb sequence in a single pass, so it adequately covers the majority of transcripts and provides the full-length nucleotide information without assembly [5,6]. SMRT presents huge advantages in transcriptomic analysis, such as the identification of novel transcripts, detection of alternatively spliced isoforms and non-coding RNA, especially in non-reference species [7]. In contrast, second-generation sequencing can read short sequences with high throughput and low cost, and it has an advantage in determining the accurate expression profiles of genes [8]. Furthermore, integrating second- and third-generation sequencing can produce comprehensive information to explore complex biological questions [7].

Environmental stimuli in plants produce various specialized metabolites. Higher levels of glucosinolates were produced in moderate and pronounced chilling conditions in *Arabidopsis* [9]. A high spermidine content was produced in chilling-treated cucumber; this metabolite also contributes to chilling tolerance [10]. Metabolites can bridge genotypes and phenotypes, and their presence and levels produce important clues to explore complex biological processes [11]. Multi-omics analysis revealed the regulatory network of steroidal glycoalkaloids in tomato fruit over the course of tomato domestication [12]. Metabolome-associated transcriptome analyses identified the putative terpene synthase genes affecting the biosynthesis of aroma-related volatile organic compounds in litchi flower [13]. The integration of transcriptome and metabolome revealed the defensive role of the spermidine decarboxylase gene *ADC1* (*Arginine decarboxylase gene*) and its associated pathway in response to cold stress in potato [14].

In contrast to the freezing sensitivity of cultivated potato, the wild relative *S. commersonii* possesses superior freezing tolerance (semi-lethal temperature is −11.5 °C or −4.5 °C with or without cold acclimation, respectively) [15]. Two major QTLs conferring freezing tolerance for this species were mapped to two intervals on chr02 and chr12 through a BC1 segregation population [16]. Furthermore, the overexpression of *ScGolS1* (*Galactinol Synthase 1*) derived from *S. commersonii* significantly increased freezing tolerance (−2 ℃) in transgenic potato [15]. These findings expanded our understanding of freezing tolerance, illustrating that multiple genes and related pathways regulate it. Therefore, a comprehensive analysis of different potato varieties with contrasting traits based on the transcriptome and metabolome will provide new insights into freezing tolerance. In this study, we analyzed *S. tubersosum* (DM for short, doubled monoploid line) and *S. commersonii* (CM for short). The flavonoid pathway responded more strongly to freezing in CM than in DM. We confirmed this result by metabolic profiling and qRT-PCR verification of major alternative splicing isoforms of *CHS*-*1* and *F3′H*-*1*. In addition, three novel transcription factors and three GTs (*Gal*-*GT*, *Man*-*GT*, *Glu*-*GT*) in the flavonoid pathway, potentially contributing to freezing tolerance, were identified.

## 2. Results

### 2.1. Full-Length Transcriptome Analysis of S. commersonii

**Figure 1:**
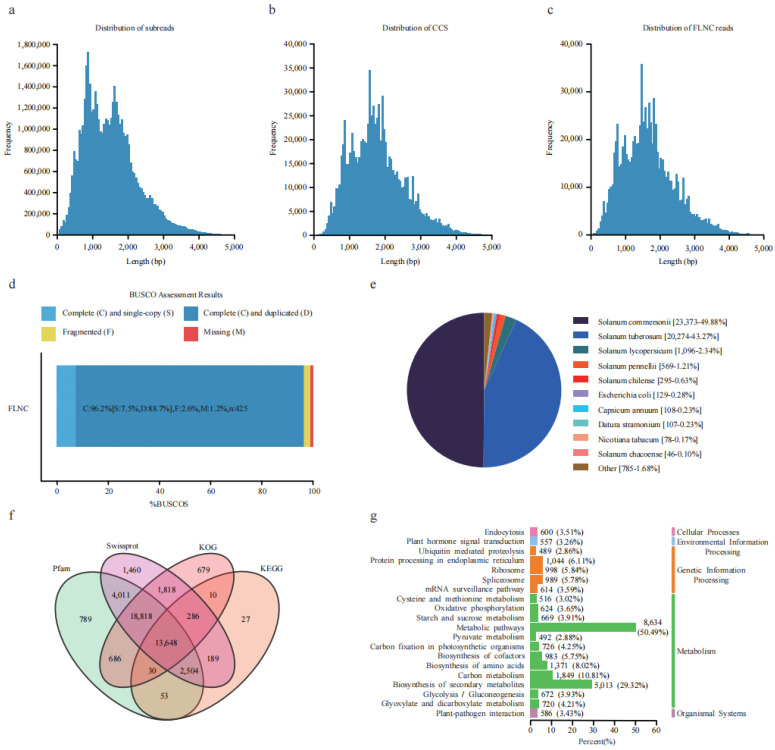
Overview of the full-length transcripts of *S. commersonii*. (**a**) Length distribution of subreads. (**b**) Length distribution of circular consensus sequences (CCS). (**c**) Length distribution of full-length non-chimeric reads (FLNC). (**d**) BUSCO evaluation of the transcript completeness. (**e**) Annotation of the full-length non-redundant isoforms from four databases. (**f**) Transcript annotation in Nr database. (**g**) KEGG functional annotation of the transcript.

A total of 71.27 Gb of data was obtained using the Pacbio Sequel platform, containing 46.94 Mb subreads with an average length of 1518 bp (Figure 1a). The self-corrected redundant subreads generated circular consensus sequences (CCS). A total of 878,975 CCS with an average length of 1711 bp were generated by merging subreads (Figure 1b). After correction and clustering, 868,272 full-length non-chimeric reads (FLNCs) with an average length of 1672 bp were obtained from the CCS reads (Figure 1c). Subsequently, FLNCs were used to generate 62,511 high-quality and 13 low-quality isoforms. The high-quality isoforms with a length in the range of 58–5506 bp were used for further study. BUSCO (Benchmarking Universal Single-Copy Orthologs) evaluation showed that 96.2% of the isoforms were complete (Figure 1d), indicating the high quality of this transcriptome data. The isoforms generated 49,232 high-quality transcripts through matching and folding, representing 12,811 gene loci.

### 2.2. Functional Annotation of High-Quality Transcripts from S. commersonii

Among all the transcripts, 46,857 (95.18%) were annotated in the NR protein database. The top three best BLAST hits were *S. commersonii* (49.88%), *S. tuberosum* (43.27%), and *S. lycopersicum* (Figure 1e). Using Swissprot, KEGG (Kyoto Encyclopedia of Genes and Genomes), Pfam (The Pfam protein families database), and KOG (Clusters of orthologous groups for complete eukaryotic genomes) for further annotation, a total of 2955, 6767, and 21,638 occurred in one, two, and three shared databases (Figure 1f). These 29,468 isoforms were grouped into three major GO categories (biological process, cellular component, and molecular function). For biological processes, the top three subgroups were the cellular process (20,875), metabolic process (18,618), and response to stimulus (12,777). For the cellular component, the top three subgroups were cell (26,433), cell part (26,433), and organelle (21,755). For molecular function, the top three subgroups were catalytic activity (13,374), binding (10,926), and transporter activity (1923) (Figure S1). In the KEGG classification, 29,451 isoforms were annotated in five branches (cellular processes, environmental information processing, genetic information processing, metabolism, and organismal systems), of which a high percentage of the isoforms (50.49%) were included in the metabolic pathways, with the second highest in the biosynthesis of secondary metabolites (Figure 1g), thus indicating that metabolic regulation plays an essential role in potato freezing responses (Table S1).

### 2.3. The Classification of Non-Redundant Transcripts

**Figure 2:**
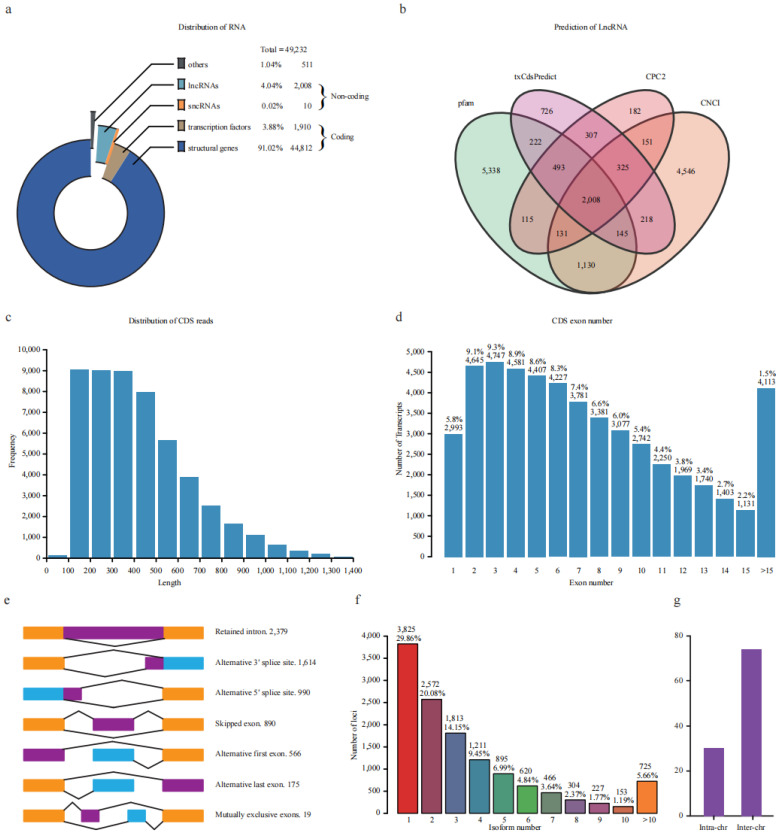
Characterization of non-redundant transcripts. (**a**) Categories of transcripts based on coding ability. (**b**) Venn diagram of the predicted lncRNA using four databases. (**c**) Length distribution of CDS reads length. (**d**) Statistic of exon numbers from one locus. The number and percentage were marked above the bars. (**e**) Visualization of seven types of alternative splicing events. The number was marked after the type. (**f**) Statistic of the isoform number produced by one locus. The number and percentage were marked above the bars. (**g**) The number of inter-/intra-chromosomal fusion transcripts.

The transcripts were classified into non-coding and coding sequences (CDS) according to their coding ability (Figure 2a). A total of 2018 shared transcripts were supported as non-coding sequences by four databases, including 2008 long non-coding RNA (lncRNA, length > 200 bp) and 10 small non-coding RNA (sncRNA, length < 200 bp) (Figure 2b). A total of 86.25% (1732) lncRNA sequences overlapped with the 2245 unannotated transcripts.

In addition, 46,772 transcripts were predicted to possess coding ability with lengths ranging from 100 to 1719 bp, with 99% of CDS being longer than 100 bp (Figure 2c). The exon number of all expressed genes was also investigated, and 75.4% of genes had less than ten exons (Figure 2d). Furthermore, the CDSs were blasted against PlantRegMap (plant regulation data and analysis platform) for transcription factor (TF) prediction. Finally, 44,812 and 1910 CDSs were annotated as structural genes and TFs, accounting for 91.02% and 3.88% of all these transcripts, respectively (Figure 2a). These putative TFs could be categorized into 54 families; the top 15 families account for 65.62% of all TFs. The most abundant family was *bHLH*, which contained 44 members, followed by *MYB*-*related* (*n* = 40), *ERF* (*n* = 36), *C3H* (*n* = 33), *C2H2* (*n* = 33), *bZIP* (*n* = 32), and *MYB* (*n* = 32) (Figure S2).

Alternative splicing (AS) is an important way for the post-transcriptional regulation of genes. Therefore, we comprehensively surveyed AS events in response to freezing stress. We found that 70.14% and 50.06% of gene loci transcripts harbor more than one and two isoforms, respectively (Figure 2f). A total of 6633 AS events corresponding to 3045 genes loci were identified. These ASs were divided into intron retention (IR, 2379), alternative 3′ splice site (A3SS, 1614), alternative 5′ splice site (A5SS, 990), skipped exon (SE, 890), alternative first exon (AF, 566), alternative last exon (AL, 175), and mutually exclusive exon (MX, 19) types, which account for 35.87%, 24.33%,14.93%, 13.42%, 8.53%, 2.64%, and 0.29% of all events, respectively (Figure 2e). Full-length transcripts were also used to identify transcript fusion events. A total of 104 were found, including 74 inter-chromosomal and 30 intra-chromosomal fusion transcripts (Figure 2g). These results revealed that full-length transcriptomic analysis could identify a high degree of alternative splicing in potato during freezing stress.

### 2.4. Differentially Expressed Genes in Response to Cold Stress

**Figure 3:**
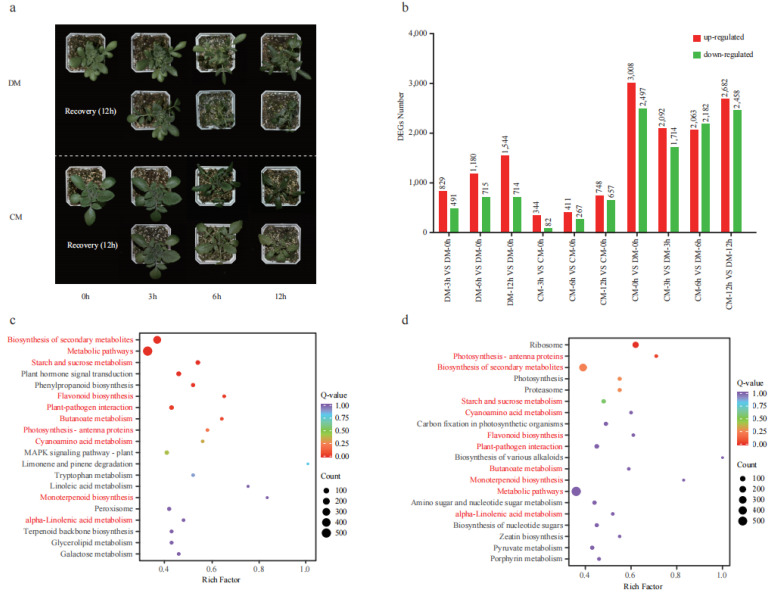
The phenotype of two varieties (DM and CM) under freezing treatment and transcriptome analysis. (**a**) The phenotype of two varieties under freezing treatment (0th, 3rd, 6th, and 12th) and recovery. (**b**) The number of differentially expressed genes (DEGs). The red and blue columns represent upregulated and downregulated genes with the number marked above, respectively. (**c**) KEGG enrichment analysis of the DEGs between two varieties at the 3rd hour. (**d**) KEGG enrichment analysis of the DEGs between two varieties at the 6th hour. Compared pathways sharing two pairs were marked in red.

The two potato varieties were treated in the freezing condition (−3 °C) for 12 h and showed distinct phenotypic responses to freezing treatment. At the 3rd hour, both varieties did not show freezing damage. At the 6th hour, DM showed obvious freezing damage with wilting leaves, while the leaves of CM were entirely frozen but not wilting. At the 12th hour, severe damage occurred on DM with a wilting stem, while mild damage occurred on CM with slightly curled leaves. After a 12 h recovery period at room temperature (25 °C), DM was completely dead, while CM could recover to a normal condition with minor injuries (Figure 3a). A comprehensive transcriptome analysis for the two varieties during this freezing stress time course was performed to reveal the regulatory mechanism of potato coping with freezing stress. Compared to the 0th hour, a total of 1320 (829 up and 491 down), 1895 (1180 up and 715 down), and 2258 (1544 and 714 down) differentially expressed genes (DEGs) were identified at the 3rd, 6th, and 12th hour in DM, respectively. A total of 426 (344 up and 82 down), 678 (411 up and 267 down), and 1405 (748 and 657 down) differentially expressed genes (DEGs) were identified at the 3rd, 6th, and 12th hour in CM, respectively (Figure 3b). Collectively, 1519 DEGs were found between any two time points in DM, and 24.22% of genes produced 957 AS events. A total of 1271 DEGs were found between any two time points in CM, and 20.38% of genes produced 589 AS events (Table S2).

Coincidentally, the number of upregulated DEGs is larger than that of downregulated DEGs in all three pairs for both varieties. This may indicate that many more positive regulatory genes than negative regulatory genes were activated in response to freezing and may also indicate that both varieties responded to freezing by activating a similar or the same mechanism. We analyzed the DEGs from each variety using KEGG, and the top 20 pathways (*p* < 0.05) were investigated. A total of 25 enriched pathways were shared between the two varieties, such as starch and sucrose metabolism, glutathione metabolism, galactose metabolism, zeatin biosynthesis, flavonoid biosynthesis, and fatty acid metabolism (Figure S3).

When we compared the DEGs between the two varieties, a total of 5505 (3008 up and 2497 down), 3806 (2092 up and 1714 down), 4245 (2063 and 2182 down), and 5140 (2682 up and 2458 down) DEGs were identified at the 0th, 3rd, 6th, 12th hour, respectively (Figure 3b). As seen in the 6th hour, DM showed obvious phenotype damage; the DEGs between the two varieties at the 3rd and 6th hour were further analyzed using KEGG (Figure 3c,d). Among the top 20 pathways, 7 were found in both time points, including starch and sucrose metabolism, flavonoid biosynthesis, plant pathogen interaction photosynthesis, cyanoamino acid metabolism, peroxisome, and alpha-linolenic acid metabolism. These results suggested that these pathways were strongly responsive to freezing and might play important roles in freezing tolerance. Given that flavonoids are reported to contribute to cold tolerance [17], we detected the contents of flavonoids in these two varieties.

### 2.5. Differentially Accumulated Metabolites in Response to Freezing Stress

**Figure 4:**
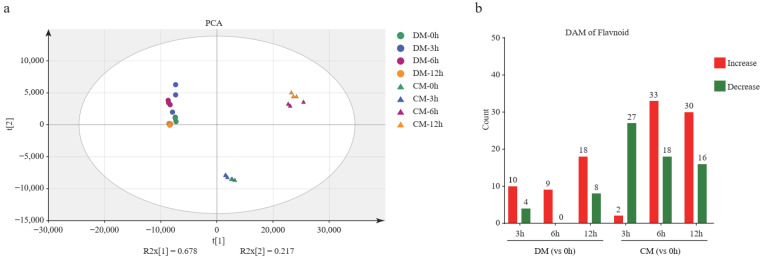

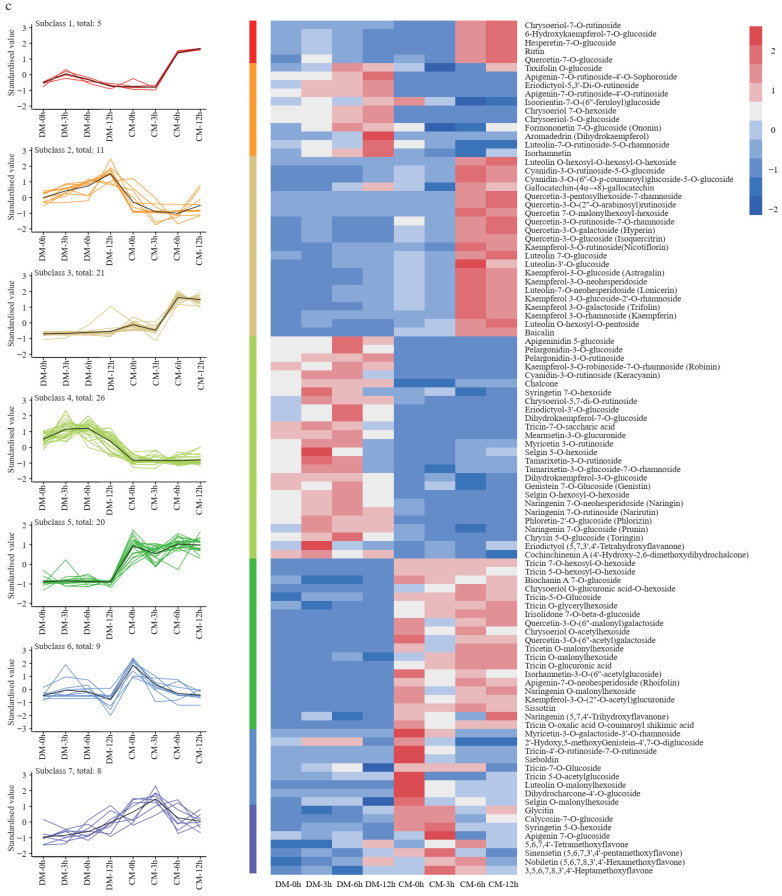
The analysis of the flavonoid metabolome. (**a**) Principal component analysis (PCA) of the flavonoid metabolites in two varieties during freezing treatment. (**b**) Differentially accumulated metabolite (DAM) analysis of two varieties during freezing treatment. (**c**) The K-means clustering analysis of DAMs and heatmap of metabolite content. The number of flavonoids in each subclass was marked with *n*.

A total of 100 flavonoid metabolites were identified, involving 9 species; they are flavone (*n* = 38), flavonol (*n* = 31), flavanone (*n* = 11), isoflavone (*n* = 8), anthocyanins (*n* = 5), dihydrochalcones (*n* = 4), chalcone (*n* = 1), flavanonol (*n* = 1), and proanthocyanidins (*n* = 1). Principal component analysis showed that all time points of DM were clustered together, indicating that the changes of metabolites in DM are not significant during freezing stress. However, the samples of CM at the 0th and 3rd hour diverged from the samples of CM at 6th and 12th, indicating that many more changed metabolites occurred. The flavonoid accumulation patterns of the resistant and sensitive potato varieties differed, and between 3 and 6 h of freezing CM illustrated a strong metabolic change, while DM did not (Figure 4a). This result indicates that the time the period between the 3rd and 6th hours is important for activating CM’s metabolic-related pathway. Furthermore, we found that 49 and 68 metabolites were significantly affected by freezing stress in DM and CM, respectively (Tables S3 and S4). The number of differentially accumulated metabolites (DAM) was 14 (10 up and 4 down), 9 (9 up), and 26 (18 up and 8 down) after freezing treatment for 3, 6, and 12 h in DM, respectively. The DAM was 29 (2 up and 27 down), 51 (33 up and 18 down), and 46 (30 up and 16 down) after freezing stress treatment for the 3, 6, and 12 h in CM, respectively (Figure 4b). In summary, many more metabolites presented significant changes in CM than those in DM. In addition, except for the 3rd vs. 0th hour, the number of accumulated metabolites was much more than the decreased metabolites in CM. This indicated that more active flavonoid regulation occurred in CM than in DM.

K-means clustering analysis was used to group the changing metabolites, producing seven subclasses. The five metabolites in Subclass1 increased to the highest level at the 3rd hour and then decreased in DM. However, in CM, they sharply increased by more than six-fold at the 6th hour and then slowly increased to the highest level at the 12th hour. The 11 metabolites in Subclass 2 steadily increased to the highest level at the 12th hour in DM, while they first decreased and then increased in CM. The 21 metabolites in Subclass 3 changed in a narrow range in DM but sharply increased to a high level at the 6th hour in CM. These metabolites mainly belong to the quercetin and kaempferol pathway. Subclass 4 includes 26 metabolites, and these metabolites first increased and then decreased in DM but essentially stayed unchanged at a low level in CM. The 20 metabolites in Subclass 5 mostly stayed unchanged at a low level in DM, but in CM, they first decreased and then increased. The nine metabolites in Subclass 6 first increased then decreased in DM but kept dropping to their lowest level in CM at the 12th hour. Finally, the eight metabolites in Subclass 7 continuously increased in DM, while in CM, they first increased to the highest level at the 3rd hour and then decreased during further freezing stress (Figure 4c).

### 2.6. The Flavonoid Pathway Was Significantly Upregulated in CM, More So than in DM

**Figure 5:**
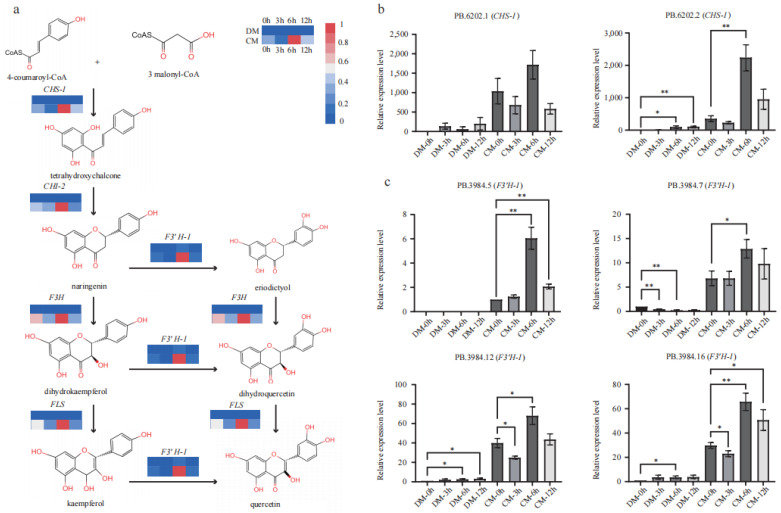
The expression pattern of major genes of the flavonoid pathway in DM and CM. (**a**) The pathway of flavonoid and the expression levels of *CHS*, *CHI*, *F3H*, *F3′H*, and *FLS* based on FPKM. (**b**) qRT-PCR identification of two *CHS* isoforms. Error bars indicate the mean ± standard error (SE) (*n* = 3). (**c**) qRT-PCR identification of four *F3′H* isoforms. Error bars indicate the mean ± standard error (SE) (*n* = 3). A two-sided *t*-test determined statistical significance. One and two stars indicate *p* < 0.05 and *p* < 0.01, respectively.

Five key genes in the flavonoid biosynthesis pathway from 4-coumaryoyl-CoA to kaempferol and quercetin via naringenin were comprehensively investigated based on transcriptomics (Figure 5a). All these genes showed increased expression in response to freezing in both varieties based on FPKM (Table S5). Specifically, the expression level of all the genes increased to the ma ximum values under freezing stress at the 6th hour. At the start of the pathway, *CHS*-*1* strongly responded to the freezing stress in both varieties. Compared to the 0th hour, the FPKM of *CHS*-*1* increased up to 12- and 3-fold in DM and CM, respectively. *CHS*-*2* increased by 3.6-fold but at a low level (FPKM < 10) in DM, while remaining relatively unchanged in CM during stress. These results indicate that *CHS*-*1* should be the predominant functional homolog, as *CHI*-*1* and *CHI*-*2* responded at different levels. *CHI*-*1* only increased by 1.5-fold in the two varieties, while *CHI*-*2* increased roughly by 6- and 2-fold in DM and CM, respectively. These results indicate that *CHI*-*2* should be the predominant functional homolog. The significantly upregulated levels of these two genes enable much more metabolic flow into the flavonoid pathway. Downstream components *F3′H*-*1* showed a maximum changing pattern, having more than 15- and 6-fold increases in DM and CM during freezing stress, respectively. *F3′H*-*2* increased 1.7-fold but at a low level (FPKM < 10) in DM, while maintaining relatively unchanged levels in CM during stress. *FLS* increased by 3- and 2-fold in DM and CM, respectively. Comparing the two varieties simultaneously, all assigned genes (*CHS-1*, *CHS-2*, *CHI-1, CHI-2*, *F3H*, *F3′H-1*, *F3′H-2* and *FLS*) (Table S5) in flavonoid pathway showed a much higher expression level in CM than those in DM.

### 2.7. qRT-PCR Confirmed the Expression Levels of CHS-1 and F3′H-1

Two isoforms (PB.6202.1 and PB.6202.2) of *CHS*-*1* were identified from the AS data, and both showed significantly upregulated expression patterns in CM more so than in DM (Table S5). qRT-PCR verified their transcript levels, and the results showed that the two transcripts were upregulated during freezing treatment in both varieties, while PB.6202.1 and PB.62020.2 showed a higher level in CM than DM at each time point (Figure 5b). qRT-PCR also indicated that PB.62020.2 might primarily regulate the flavonoid metabolome under freezing stress. Based on the AS data, *F3′H*-*1* produced seven major isoforms (filtered with FPKM > 2). Four of them, PB3984.5, PB3984.7, PB3984.10, and PB3984.16, were unique in CM, and the other three isoforms showed a much higher expression in CM than in DM (Table S5). These four transcripts, PB3984.5, PB3984.7, PB3984.12, and PB3984.16, were verified by qRT-PCR (Figure 5c; Table S6). In agreement with the unique expression of PB3984.5 in CM based on FPKM, its expression was only detected in CM and increased to the max level under freezing stress at the 6th hour. All the rest of the three transcripts showed a much lower relative expression level in DM, while in CM, they showed high relative expression levels, especially at the 6th hour. The transcriptome analysis and qRT-PCR results showed that the major structural genes in the flavonoid pathway were significantly upregulated in CM under freezing stress. These extremely higher expression levels might contribute to the strong frost tolerance of *S. commersonii*.

### 2.8. Multi-Omics Reveals New Functional Genes in the Flavonoid Pathway

**Figure 6:**
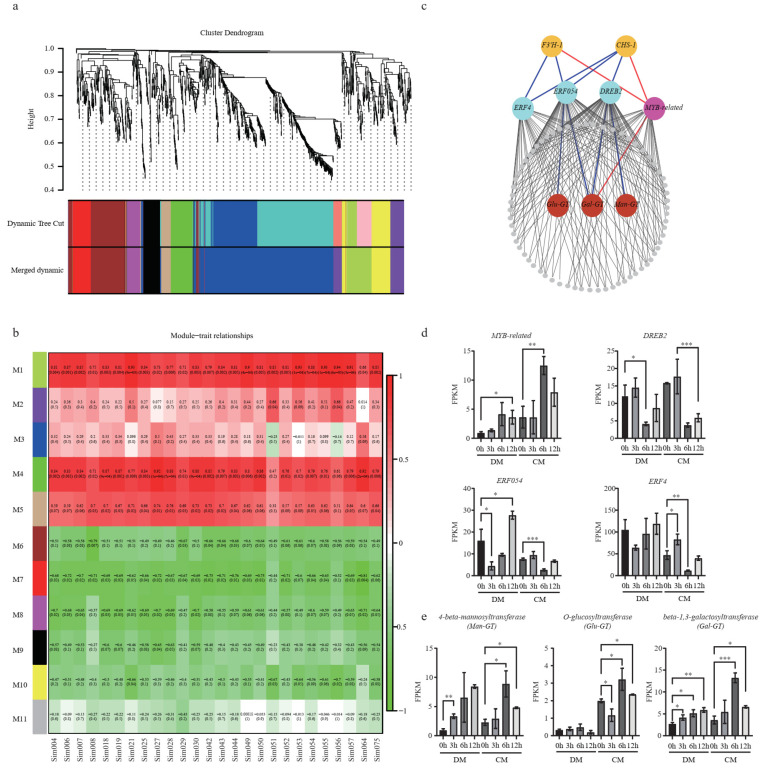
Co-expression network analysis identified new freezing-responsive genes in the flavonoid pathway. (**a**) Hierarchical clustering tree of the modules. (**b**) Module–trait relationships calculated by Pearson correlation coefficients. (**c**) The co-expression network of M1. The triangle and circle strands for TFs and structural genes, respectively. The red and blue lines indicate the positive and negative correlation, respectively; (**d**). Expression levels of *MYB*-*related*, *DREB2*, *ERF054*, and *ERF4*. (**e**) *Man*-*GT*, *Glu*-*GT*, and *Gal*-*GT* in CM and DM under freezing stress at different time points. A two-sided *t*-test determined statistical significance. One, two and three stars indicate *p* < 0.05, *p* < 0.01 and *p* < 0.001, respectively.

In order to comprehensively reveal the regulatory network of flavonoids subjected to freezing stress (Figure 6a), 1271 DEGs from CM were used for co-expression analysis. A total of 10 modules (except for M11) were identified, containing from 38 to 496 genes. To bridge the transcriptome and metabolome, we performed a correlation analysis between the gene modules and DAMs. Two modules, M1 and M4, were identified based on the correlation coefficients (*r* > 0.7 and *p* < 0.05) (Figure 6b). Interestingly, both *CHS*-*1* and *F3′H*-*1* were grouped in module M1. We next used these two genes as baits to search for possible regulators and isolated one *Myb*-*related* and three *ERFs* (*DREB2*, *ERF054*, and *ERF4*) (Figure 6c). Their expression levels during freezing treatment were confirmed by the FPKM value (Figure 6d). They were strongly responsive to freezing treatments. Compared to the 0th hour in CM, the expression of *Myb-related* reached the highest value at the 6th hour, while *DREB2*, *ERF054*, and *ERF4* were upregulated at the 3rd hour and then downregulated at the 6th hour. Using these TFs as baits, three UDP-transferases, *β*-*1,3*-*galactosyltransferase* (*Gal*-*GT*), *4*-*beta*-*mannosyltransferase* (*Man*-*GT*), and *O*-*glucosyltransferase* (*Glu*-*GT*)*,* were identified. *Gal*-*GT* was simultaneously highly related to *MYB-related, ERF054*, and *DREB2*. *Man*-*GT* and *Glu*-*GT* were highly negatively related to *DREB2* and *ERF054*, respectively (Figure 6c). Compared to the normal condition, in CM, the expression levels of *Gal*-*GT*, *Man*-*GT*, and *Glu*-*GT* were upregulated by 3.7-fold, 3.8-fold, and 1.6-fold at the 6th hour, respectively (Figure 6e; Table S5). Using correlation analysis between these three GTs and all the glycosylated flavonoids, we found a total of 8, 4, and 5 glycosylated flavonoids, which are highly correlated (|R| > 0.8, *p* < 0.05) with these three genes, respectively, indicating that these identified GTs very likely play important roles in the glycosylation of flavonoids to protect against plant freezing stress (Table S7).

## 3. Discussion

During the long process of evolution, plants have generated multiple strategies to cope with stimuli in cold environments. The transcriptional regulation of co-responding genes is one important way to deal with cold stress. Multiple pathways are activated or suppressed in this process to alleviate cold damage. Here, we viewed that many genes and pathways dramatically respond to freezing treatment. A total of 1519 and 1271 genes were responsive to freezing stress in the two varieties examined. These genes were enriched in 25 shared pathways for both varieties (Figure S3). For example, starch and sucrose metabolism were found in three pairs of time-point comparisons, indicating its important role in coping with freezing in potato.

As an osmotic regulator and penetration protectant, sugar can reduce the cell’s water potential and protect and maintain membrane stability. In chill-treated maize, *raffinose synthase* (*RAFS*), the key enzyme for raffinose biosynthesis, was significantly upregulated, and its photosynthetic product sugar was significantly accumulated. Seedings with high raffinose accumulation exhibited better chilling stress tolerance [18]. In *Camellia sinensis*, the content of soluble sugars in leaves rose under low-temperature stress [19]. Both the biosynthesis of unsaturated fatty acids and fatty acid elongation pathways were found in two pairs of time-point comparations in chickpea. The unsaturated fatty acid accumulation of membrane lipids was correlated with cold stress resistance. As well, after cold acclimation, there was an increase in the unsaturated fatty acid ratio compared with the saturated ones [20]. A study of winter wheat showed that linolenic acid and palmitic acid were closely related to cold resistance, and the index of unsaturated fatty acid variation is increased in a cold tolerance genotype [21]. In the cold-tolerant genotype *S. commersonii*, stearoyl-ACP (acyl carrier protein) desaturase was activated after cold acclimation and high polyunsaturated fatty acids were highly accumulated in the leaf [22]. Plant hormone signal transduction occurred at five time-point comparisons. Plants can regulate hormones in answer to cold stress, and these hormones can also aid in stress protection. Maize seedlings pretreated with ABA present greater cold tolerance than untreated seedlings [23]. Undoubtedly, these identified enriched pathways provide many clues for unraveling the freezing tolerance mechanism in potato.

Low temperatures can produce ROS (reactive oxygen species) accumulation, which affects many cellular functions [24]. Plants can protect against outer stress by activating antioxidant mechanisms for scavenging ROS [25], including enzymatic and non-enzymatic antioxidants. Flavonoids are among the most bioactive plant non-enzymatic antioxidants and are reported to perform better than other antioxidants, such as ascorbate [26]. *S. commersonii* presents superior freezing tolerance and could contribute excellent alleles for anti-frost breeding in potato. A few studies have been conducted to explore its freezing tolerance mechanism under different aspects, including soluble carbohydrate synthesis and *CBF* (*C*-*repeat binding factor*) regulons expression [27,28]. However, the flavonoid pathway contribution to the anti-frost trait of CM was never explored. In this study, we found that flavonoid biosynthesis genes were strongly responsive to freezing in both varieties, and correspondingly, the flavonoid content was affected by freezing treatment.

Compared with DM, more (68% vs. 49%) flavonoids showed significant change in CM, and these metabolites presented different, changing patterns, such as the Subclasses 1, 3 and 5, including 46 metabolites, which present high accumulation in CM but not in DM. Coincidentally, all detected quercetin (*n* = 9) and 7 out of 8 Kaempferol-3-glycosyl were among them. In a previous study, the effect of flavonoid content on freezing tolerance was carried out using 20 *Arabidopsis* mutant lines. All the knock-out mutants of flavonoid biosynthesis genes had reduced flavonoid content and showed impaired leaf-freezing tolerance. In contrast, the *MYB* activation mutant line pap1-D accumulated a high content of quercetin-3Glc, Kaempferol-3Rha-7Rha, and three anthocyanins, and showed improved freezing tolerance [24]. This study demonstrates the contribution of flavonoids to freezing tolerance, especially certain flavonoids such as glycosylated flavonoids.

Corresponding to the accumulation pattern of metabolites in CM, nearly all the major structural genes were significantly upregulated in CM, especially at the 6th hour after freezing (Figure 5), while these genes only showed a mild upregulation in DM. In addition, all the AS isoforms in CM showed an overwhelming expression superiority to that in DM. An upregulated pathway means an upstream regulator was also activated or suppressed. Four TFs were identified based on gene expression correlation. The *MYB*-*related* gene showed a high positive correlation (R > 0.9) with *CHS*-*1* and *F3′H*-*1*, indicating it may activate these structural genes. *FtMYB12* in *F. tataricum* could be greatly induced by low temperatures, and its overexpression in *Arabidopsis* resulted in enhanced cold tolerance [29]. *AtMYB12* could upregulate a related gene and lead to the production of rutin in transgenic tobacco callus cultures [30]; rutin is also a glycosylated flavonol. Meanwhile, three *ERF* genes were also identified. Multiple studies found that *ERFs* could be induced by cold and were key transcription factors involved in cold-responsive gene expressions [31,32,33]. In addition, several studies revealed that some *ERFs* could regulate flavonoid biosynthesis. In citrus, *CitERF32* and *CitERF33* could activate the transcription of *CHI*, affecting the accumulation of flavonoid production [34]. In tomato, the overexpressing *SlERF.G3-like* could increase the expression of structural genes and lead to the accumulation of flavonoid compounds [35]. In addition, multiple studies found that cold could induce *ERFs* and were the key transcription factors involved in cold-responsive gene expressions. Although the three identified *ERFs* negatively correlated with *CHS*-*1* and *F3′H*-*1* from the network, their expression level increased (3rd vs. 0th) in CM.

In plants, flavonoids usually exist in their “decorated” forms catalyzed by different enzymes, such as *glycosyltransferases* or *methyltransferases*. The glycosylation of flavonoids usually confers the biological activity of corresponding derivatives [36]. For example, in *Arabidopsis*, two *UDP-glycosyltransferases* could be induced by cold, salt, and drought stresses, and they could modulate the accumulation of modified anthocyanin, contributing to enhanced stress tolerance [37]. In rice, two *UDP-glycosyltransferases* affect the natural variation in flavones, and product accumulation contributes to UV-B tolerances [36]. Here, three UGTs were found to be related to flavonoid biosynthesis and freezing stress. However, different UGTs show different substrate specificity, and the end products can also present different biological activity. Therefore, further experimental studies are needed to clarify their exact functions.

## 4. Materials and Methods

### 4.1. Plant Material and Sample Preparation

The plant varieties used in this study were *Solanum tubersosum* (DM) and *Solanum commersonii* (CM). The plants were grown for 30 days under a 16 h light/8 h dark photoperiod, placed for 3 days at 3 °C followed by −3 °C treatment, and leaf samples were harvested at 0 h, 3 h, 6 h, and 12 h for transcriptome and metabolome assays. Three biological replicates for each time point were prepared. Total RNA was isolated from the above samples using RNAprep Pure Polyphenol Plant TOTAL RNA Extraction Kit (DP441) and treated with DNase I (Fermentas, Carlsbad, CA, USA) according to the manufacturer’s instructions. The RNA from each sample was sequenced using 150-bp paired-end Illumina sequencing with libraries of 350-bp insert sizes. In addition, one bulked RNA sample of *S. commersonii*, generated from all time points, was sequenced using the Pacbio Sequel platform.

### 4.2. Illumina Data Analysis

Fragments were mapped to the reference genome sequence (DM_v6.1) using hisat2. The gene expression levels were estimated by FPKM (fragments per thousand bases of transcript per million mapped reads). Differential expression analysis was performed using the DESeq R package. Genes identified by DESeq with *padj* (*q*) < 0.05 and Log2 (Fold-Change) ≥ 1 or Log2 (Fold-Change) ≤ −1 were defined as differentially expressed. The gene expression profiles were calculated using StringTie v.1.3.3.

### 4.3. PacBio Data Analysis

The Iso-Seq data were processed using SMRT-Analysis software package v3.0 (https://github.com/ben-lerch/IsoSeq-3.0/blob/master/README.md, accessed on 19 January 2022). A circular consensus sequence (CCS) was generated from subread BAM files with the following parameters: skip-polish, min-passes 2, and min-rq 0.99. CCS reads were classified into full-length and non-full-length reads using lima. Full-length FASTA files were fed into the cluster and polish step with default parameters. The polished reads were aligned to the DM genome (v6.1) using the software pbmm2 (https://github.com/PacificBiosciences/pbmm2, accessed on 19 January 2022). Finally, the aligned BAM files were fed into the collapse step with default parameters. BUSCO was used to evaluate the quality of full-length transcripts. AS events were extracted and quantified using SUPPA2 (https://github.com/comprna/SUPPA, accessed on 21 January 2022) based on a GFF containing long-read isoforms. TransDecoder (https://github.com/TransDecoder/TransDecoder/releases, accessed on 22 January 2022) was used to identify CDS regions within the transcript sequences. For transcription factor (TF) prediction, we first detected the ORFs of isoforms by TransDecoder. Then, the ORFs were mapped onto PlantTFDB (http://planttfdb.gao-lab.org/prediction.php, accessed on 23 January 2022).

### 4.4. Identification of the lncRNA

CNCI (Coding-Non-Coding Index), CPC (Coding Potential Calculator), txCdsPredict, and Pfam databases were used to predict the coding potential of transcripts. Transcripts with coding potential were filtered out, and those without coding potential were candidate sets of lncRNAs according to the threshold: CPC threshold < 0, CNCI threshold < 0, and txCdsPredict threshold < 500.

### 4.5. Identification of Fusion Transcripts

The Python script (fusion_finder.py) in the PBTRANSCRIPT-TOFU package (http://github.com/PacificBiosciences/cDNA_primer/, accessed on 25 January 2022) was used to identify fusion transcripts. Each candidate fusion transcript must be mapped to two or more loci with a distance >= 10 kb. Each locus must cover at least 5% of the transcripts, and the total coverage of fusion transcripts is at least 99%.

### 4.6. Functional Annotation of Genes

Genes were annotated by public databases, including the NCBI Non-Redundant Protein Database (Nr), NCBI Non-Redundant Nucleotide Database (Nt), SwissProt, Protein Family (Pfam), Gene Ontology (GO), Kyoto Encyclopedia of Genes and Genomes (KEGG), and Clusters of Orthologous Groups of proteins (KOG/COG/eggNOG). The similarity was filtered with an E-value threshold of 10^−5^.

### 4.7. Analysis of Differentially Expressed Transcripts

All Illumina reads were mapped to the SMRT full-length transcripts to identify fragments per kilobase of exon per million fragments mapped (FPKM) values. DESeq2v1.4.5 was used for differentially expressed genes (DEGs) analysis. The *p*-value could be assigned to each gene and adjusted by the Benjamini and Hochberg approach for controlling the false discovery rate. Genes with q ≤ 0.05 and |Log2 (Fold-Change)| ≥ 1 were identified as DEGs.

### 4.8. Co-Expression Analysis

A weighted gene co-expression network analysis was performed using the WGCNA package (v1.69) with the default parameters. An adjacency matrix was constructed and converted into a topological overlap matrix (TOM). A dynamic hybrid tree-cut algorithm (the R package dynamicTreeCut, v.1.63) was used to detect the modules. A correlation matrix between phenotypes and gene modules was constructed using the WGCNA package. Finally, the relationship between genes was visualized using Cytoscape (v.3.8.2).

### 4.9. Quantitative Real-Time PCR (qRT-PCR) Analysis

For RNA extraction, we used the Plant Total RNA Kit (Tiangen, Beijing, China), and PrimeScript™ RT Reagent Kit with gDNA Eraser (TaKaRa, Kusatsu, Japan) was used for the synthesis of the first-strand cDNA. The ABI Step, One Plus instrument was used for qRT-PCR experiments. Experiments were repeated 3 times. The comparative 2−ΔΔCT (A method of qRT-PCR fluorescence quantitative data analysis, ΔΔCt = ΔCt experimental group − ΔCt control group) method was used for gene expression level analysis. For quantitative analysis, Exocyst complex component gene *(Sec3A*) was used as the internal reference. Reactions contained the following: 5 μL of 2x × TB Green Premix Ex Taq II, 1 μL of template cDNA, 0.4 μL of forward and Universal miRNA qPCR Primer, 0.2 μL of Passive Reference Dye and water to 10 μL. PCR amplification was performed as follows: 95 °C for 30 s, 40 cycles of 95 °C for 5 s, 58 °C for 30 s.

## 5. Conclusions

In this study, we reconstructed a multi-omics database for freezing research in potato. KEGG enrichment analysis of DEGs revealed that some shared pathways were activated in freezing sensitive and tolerant varieties, indicating they launched similar mechanisms to cope with freezing. The transcriptome and metabolome integration illustrated that the flavonoid pathway was strongly responsive to freezing stimuli in both genotypes but to different degrees. The four newly identified transcription factors (one *Myb*-*related* and three *ERF*) and their associated UGTs are potential contributors to the freezing tolerance of CM in concert with other structural genes in the flavonoid pathway. All the results provide insights to explore the freezing tolerance mechanism mediated by the flavonoid pathway and other pathways. These results will also provide new genetic modules from *S.commersonii* for potato anti-freezing breeding.

## Data Availability

Data recorded in the current study are available in all tables of the manuscript.

## Supplementary Materials

The following supporting information can be downloaded at: https://www.mdpi.com/article/10.3390/plants12102054/s1, Figure S1: GO functional annotation of all the transcripts; Figure S2: Transcription factor family classification; Figure S3: KEGG pathway enrichment analysis of the DEGs identified in DM and CM; Table S1: Functional annotation of all transcripts; Table S2: DEGs in DM and CM under freezing treatment; Table S3: Detection of flavonoid metabolites in DM and CM; Table S4: Differential accumulated metabolites at different time points; Table S5: The expression level of *CHI*, *CHS*, *F3H*, *F3′H*, *FLS*, three *UDP—glycosyltransferases*, and four transcriptional; Table S6: Primers for quantitative PCR in DM and CM; Table S7: Correlation between different *glycosyltransferases* and glycosylated flavonoid derivatives.

## Abbreviations

SMRT	Single-molecule real-time
CCS	Circular consensus sequences
FLNC	The full length is not chimeric
AS	Alternatively spliced
TF	Transcription factor
ADC1	Arginine decarboxylase gene
DEG	Differentially expressed gene
DAM	Differentially accumulated metabolite
GO	Gene ontology
KEGG	Kyoto Encyclopedia of Genes and Genomes
Pfam	The Pfam protein family database
KOG	Clusters of orthologous groups for complete eukaryotic genomes
F3′H	Flavonoid 3′-hydroxylase
F3H	Flavanone 3-hydroxylase
CHS	Chalcone synthase
CHI	Chalcone isomerase
FLS	Flavonol synthase
