# Supplementary material for: Integrated Full-Length Transcriptome and Metabolome Profiling Reveals Flavonoid Regulation in Response to Freezing Stress in Potato
Source: Plants (Basel). 2023 May 22;12(10):2054. doi: 10.3390/plants12102054 (PMC10223400; doi:10.3390/plants12102054)
Supplement: Supplementary file 1 [file plants-12-02054-s001.zip › plants-2321875--supplementary figures.pdf]

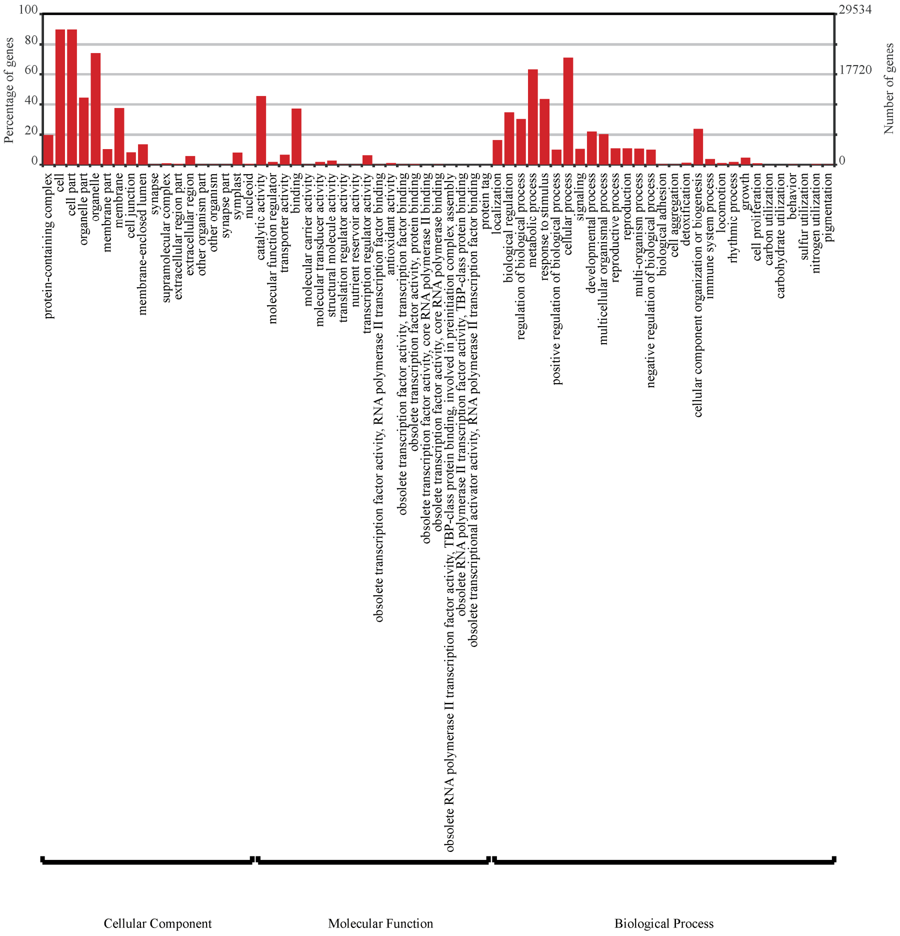

Figure S1. GO functional annotation of all the transcripts.

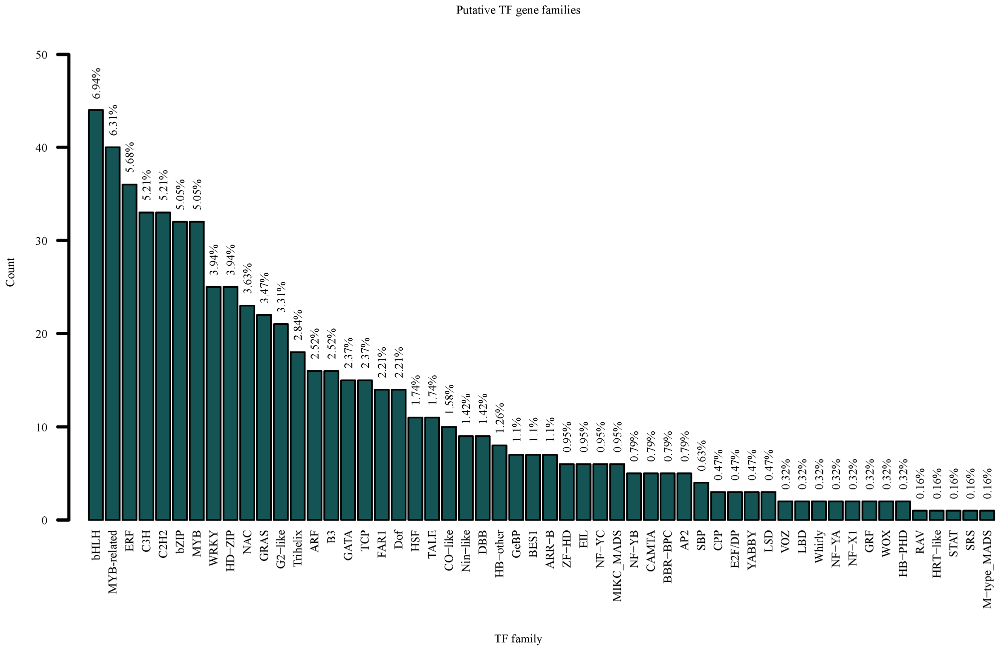

Figure S2. Transcription factor family classification.

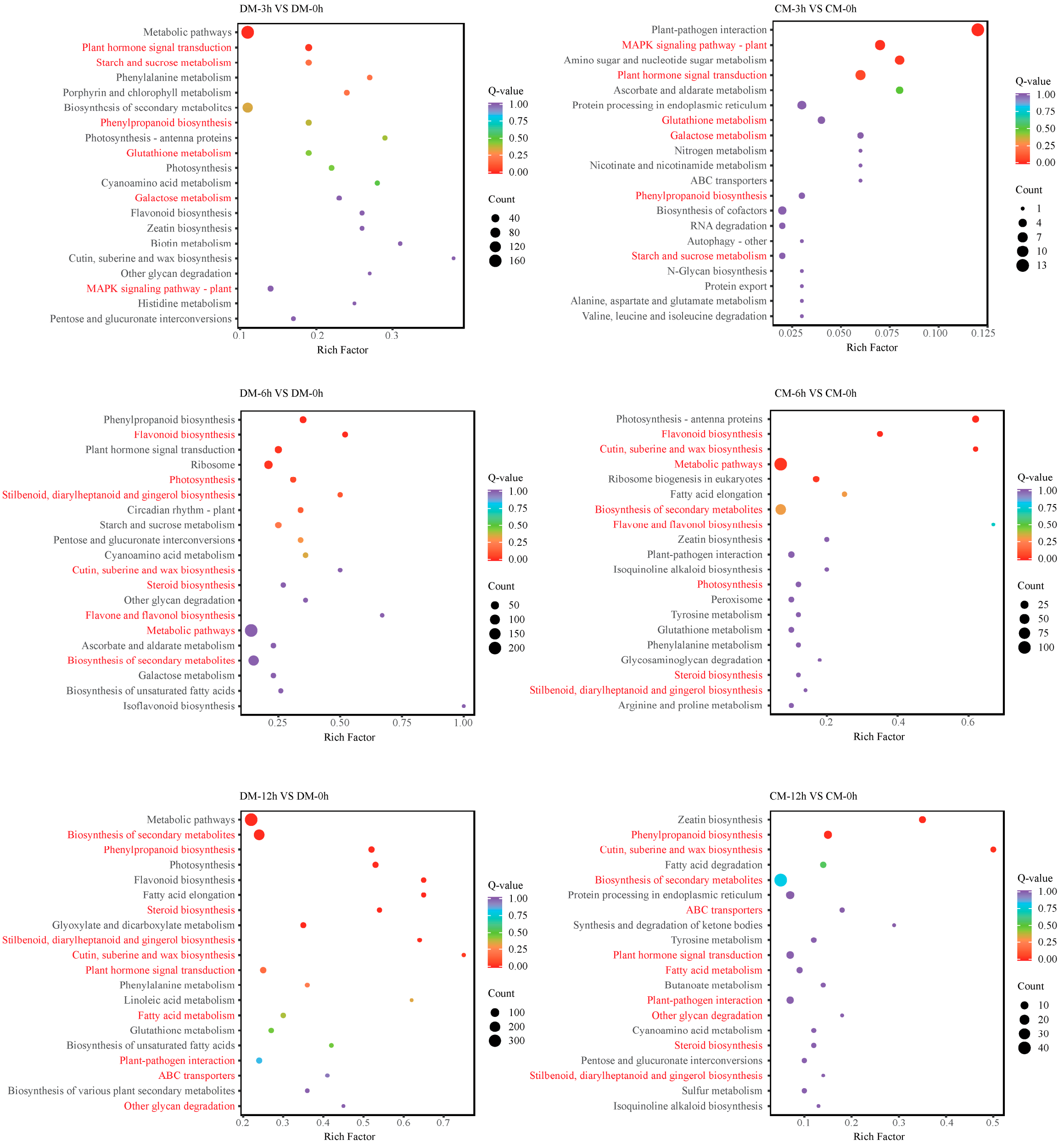

Figure S3. KEGG pathway enrichment analysis of the DEGs identified in DM and CM
